# Circ_CLASP2 Regulates High Glucose-Induced Dysfunction of Human Endothelial Cells Through Targeting miR-140-5p/FBXW7 Axis

**DOI:** 10.3389/fphar.2021.594793

**Published:** 2021-03-11

**Authors:** Qin Zhang, Jing Long, Nannan Li, Xuelian Ma, Lisheng Zheng

**Affiliations:** ^1^ Department of Cardiovascular, Dongying People’s Hospital, Dongying, China; ^2^ Department of Critical Care Medicine, Dongying People’s Hospital, Dongying, China; ^3^ Department of Clinical Laboratory, Dongying People’s Hospital, Dongying, China

**Keywords:** high glucose, circ_CLASP2, miR-140-5p, Fbxw7, ECs dysfunction

## Abstract

Hyperglycemia exposure results in the dysfunction of endothelial cells (ECs) and the development of diabetic complications. Circular RNAs (circRNAs) have been demonstrated to play critical roles in EC dysfunction. The current study aimed to explore the role and mechanism of circRNA CLIP–associating protein 2 (circ_CLASP2, hsa_circ_0064772) on HG-induced dysfunction in human umbilical vein endothelial cells (HUVECs). Quantitative real-time polymerase chain reaction (qRT-PCR) was used to assess the levels of circ_CLASP2, miR-140-5p and F-box, and WD repeat domain-containing 7 (FBXW7). The stability of circ_CLASP2 was identified by the actinomycin D and ribonuclease (RNase) R assays. Cell colony formation, proliferation, and apoptosis were measured by a standard colony formation assay, colorimetric 3-(4, 5-dimethylthiazol-2-yl)-2, 5-diphenyl-2H-tetrazolium bromide (MTT) assay, and flow cytometry, respectively. Western blot analysis was performed to determine the expression of related proteins. Targeted correlations among circ_CLASP2, miR-140-5p, and FBXW7 were confirmed by dual-luciferase reporter assay. High glucose (HG) exposure downregulated the expression of circ_CLASP2 in HUVECs. Circ_CLASP2 overexpression or miR-140-5p knockdown promoted proliferation and inhibited apoptosis of HUVECs under HG conditions. Circ_CLASP2 directly interacted with miR-140-5p via pairing to miR-140-5p. The regulation of circ_CLASP2 overexpression on HG-induced HUVEC dysfunction was mediated by miR-140-5p. Moreover, FBXW7 was a direct target of miR-140-5p, and miR-140-5p regulated HG-induced HUVEC dysfunction via FBXW7. Furthermore, circ_CLASP2 mediated FBXW7 expression through sponging miR-140-5p. Our current study suggested that the overexpression of circ_CLASP2 protected HUVEC from HG-induced dysfunction at least partly through the regulation of the miR-140-5p/FBXW7 axis, highlighting a novel therapeutic approach for the treatment of diabetic-associated vascular injury.

## Highlights

1. Circ_CLASP2 overexpression promoted proliferation and inhibited apoptosis of HUVECs under HG conditions.
2. Circ_CLASP2 directly interacted with miR-140-5p via pairing to miR-140-5p.
3. Circ_CLASP2 mediated FBXW7 expression through sponging miR-140-5p.

## Introduction

Endothelial cells (ECs) are a type of simple squamous cells that form the inner lining of blood vessels, which control the infiltration of blood cells and proteins into the vessel wall (Dejana, 2004; dela Paz and D’Amore, 2009). Chronic exposure to hyperglycemia can lead to the dysfunction of ECs and thus contributes to the development of diabetic complications, including cardiovascular disease (Ceriello, 2005; Funk et al., 2012). Hence, it is very crucial to identify effective biomarkers for the detection of ECs dysfunction and the prevention of macrovascular complications. One promising biomarker is based on circular RNAs (circRNAs) (Shang et al., 2018; Jin et al., 2019).

CircRNAs are a novel family of noncoding RNAs that lack a 5′ cap structure and a 3′ poly(A) tail (Chen and Yang, 2015). Up to now, some circRNAs have been proposed to act as critical regulators in gene expression via functioning as microRNAs (miRNAs) sponges (Granados-Riveron and Aquino-Jarquin, 2016). The abnormal expression and role of circRNAs in ECs dysfunction induced by high glucose (HG) have been widely investigated (Liu et al., 2017; Pei et al., 2018; Shang et al., 2018; Jin et al., 2019; Zhang and Sui, 2020). For example, Cheng et al. highlighted that has_circ_0068087 was highly expressed in HG-triggered human umbilical vein endothelial cells (HUVECs) and regulated HG-induced cell dysfunction by acting as an miR-197 sponge (Cheng et al., 2019). Wei et al. demonstrated that circRNA vascular endothelial growth factor C (circVEGFC) promoted HG-induced apoptosis in HUVECs through the regulation of miR-338-3p (Wei et al., 2020). Pan et al. uncovered that has_circ_0054633 protected HUVECs against HG-induced dysfunction by modulating heme oxygenase-1 (HO-1) expression via sponging miR-218 (Pan et al., 2018). Cao and colleagues reported that the overexpression of circRNA homeodomain-interacting protein kinase 3 (circHIPK3) alleviated HG-induced HUVEC impairment by sponging miR-124 (Cao et al., 2018). Recent evidence has demonstrated that circRNA CLIP-associating protein 2 (circ_CLASP2, hsa_circ_0064772) is significantly downregulated in HG-induced HUVECs (Jin et al., 2019). However, the function and mechanism of circ-CLASP2 on HG-induced HUVEC dysfunction remain to be elucidated.

MiRNAs have been illuminated to serve as essential regulators in diabetes-associated endothelial dysfunction (Arunachalam et al., 2016; Jansen et al., 2016; Bammert et al., 2017). It was reported that miR-140-5p level was elevated in endothelial progenitor cells with metabolic syndrome (Liu et al., 2016). Moreover, miR-140-5p expression was found to be upregulated in HUVECs under HG conditions, suggesting its involvement in endothelial impairment (Silambarasan et al., 2016). Nevertheless, the influence of miR-140-5p in HG-induced HUVEC dysfunction is still unknown.

In our current research, we undertook to determine the expression of circ_CLASP2 in HUVECs under HG conditions and observed the function of circ_CLASP2 in HG-induced HUVEC dysfunction. Consequently, we investigated whether the miR-140-5p/F-box and WD repeat domain-containing 7 (FBXW7) axis was a molecular mediator of circ_CLASP2 on HG-induced HUVEC impairment.

## Materials and Methods

### Cell Culture and Treatment

In this study, HUVECs (American Type Collection Culture, ATCC, Manassas, VA, United States) were used. HUVECs were maintained in endothelial cell growth medium (ECGM, Promocell, Corning, NY, United States) containing 10% fetal bovine serum (FBS, Promocell) and 1% penicillin/streptomycin (Sigma-Aldrich, Buchs, Switzerland) at 37°C, 5% CO_2_. The cells were grown to ∼60% confluence on 24-well plates and then exposed to 25 mM of d-glucose (Sigma-Aldrich) for 12, 24, and 48 h. Control wells were treated with 5 mM of d-glucose.

### Quantitative Real-Time Polymerase Chain Reaction

Total RNA was isolated from HUVECs using the QIAzol lysis reagent (Qiagen, Courtaboeuf, France) and miRNeasy kit (Qiagen) based on the guidance of producers. The synthesis of cDNA was implemented with 400 ng of RNA extracts by using the QuantiTect reverse transcription (RT) kit (Qiagen) for circ_CLASP2 and mRNAs expression or miScript II RT kit (Qiagen) for miR-140-5p analysis. The qRT-PCR was carried out on a LightCycler (Roche, Basel, Switzerland) using the Light-Cycler 480 SYBR Green master mix (Roche) as recommended by the manufacturers. The primer sequences were listed in Table 1. The PCR cycling program consisted of 95°C for 10 min and 40 cycles of 95°C for 20 s, followed by 60°C for 1 min. Results were normalized to the housekeeping gene β-actin or U6 snRNA and calculated by the 2^−ΔΔCt^ method.

**TABLE 1:** Primers sequences for PCR.

	Primers for PCR (5′-3′)	
Circ_CLASP2	Forward	CCG​GGT​GAG​AGC​AAA​ACT​TT
	Reverse	TGC​AGC​TGA​TGA​TGG​CCT​AT
CLASP2 linear mRNA	Forward	CCG​TGA​GGA​GAC​GTT​CAT​TT
	Reverse	CTT​TCT​TGC​CTC​CAC​TCT​GG
FBXW7	Forward	AAA​GAG​TTG​TTA​GCG​GTT​CTC​G
	Reverse	CCA​CAT​GGA​TAC​CAT​CAA​ACT​G
β-actin	Forward	GGC​GGA​CTA​TGA​CTT​AGT​TG
	Reverse	AAA​CAA​CAA​TGT​GCA​ATC​AA
miR-140-5p	Forward	TGC​GGC​AGT​GGT​TTT​ACC​CTA​TG
	Reverse	CCA​GTG​CAG​GGT​CCG​AGG​T
U6 snRNA	Forward	CTCGCTTCGGCAGCACA
	Reverse	AAC​GCT​TCA​CGA​ATT​TGC​GT

### Actinomycin D and Ribonuclease R Assays

For actinomycin D assay, HUVECs at ∼60% confluence were treated with 2 mg/ml of actinomycin D (Sigma-Aldrich) for 0, 6, 12, and 24 h. For RNase R digestion assay, total RNA (5 μg) was incubated in 20 μL of reaction system containing 10 U of RNase R (Epicentre, Madison, WI, United States) for 30 min at 37°C. In both assays, the levels of circ_CLASP2 and CLASP2 linear mRNA were evaluated by qRT-PCR as above.

### Cell Transfection

The synthetic miR-140-5p mimic (5′-CAG​UGG​UUU​UAC​CCU​AUG​GUA​G-3′) and negative control miR-NC mimic (5′-UUC​UCC​GAA​CGU​GUC​ACG​UUU-3′), siRNA targeting FBXW7 (si-FBXW7, 5′-ACA​GGA​CAG​UGU​UUA​CAA​A-3′) and siRNA-negative control (si-NC, 5′-UCA​CCC​AGA​UGC​CGC​UAU-3′), the inhibitor of miR-140-5p (anti-miR-140-5p, 5′-CUA​CCA​UAG​GGU​AAA​ACC​ACU​G-3′), and a scrambled control sequence (anti-miR-NC, 5′-CAG​UAC​UUU​UGU​GUA​GUA​CAA-3′) were obtained from GenePharma (Shanghai, China). Circ_CLASP2 sequence and a nontarget control sequence were synthesized by GenePharma and individually inserted into the pCD5-ciR vector (Geenseed, Guangzhou, China) opened with EcoR I and BamH I sites to generate circ_CLASP2 overexpression plasmid (circ_CLASP2) and negative control plasmid (Vector). HUVECs (1 × 10^5^ cells/well) were seeded into 24-well plates 24 h before transfection with 30 nM of the indicated oligonucleotides or/and 100 ng of plasmids using Lipofectamine 3000 (Thermo Fisher Scientific, Hemel Hempstead, United Kingdom) following the suggestion of the manufacturers. Transfected cells were harvested for expression analysis after 48 h and were exposed to HG for 24 h.

### Cell Colony Formation, Proliferation, and Apoptosis Assays

Cell colony formation and proliferation of HUVECs after various treatments were gaged as previously reported (Meng et al., 2015). For colony formation analysis, approximately 100 treated HUVECs were plated into 6-well plates and cultured under standard protocols. After 2 weeks, the cells were stained with 0.1% crystal violet and the number of colonies (>50 cells) was manually counted at five random fields. For cell proliferation analysis, about 2 × 10^3^ treated HUVECs were seeded into 96-well plates and cultured using standard protocols. On day 1, 2, and 3, a colorimetric 3-(4, 5-dimethylthiazol-2-yl)-2, 5-diphenyl-2H-tetrazolium bromide (MTT) assay was performed for the evaluation of cell proliferation based on the recommendations of producers (Solarbio, Beijing, China). Absorbance at 490 nm was measured by the Synergy MX microplate (BioTek, Bad Friedrichshall, Germany). Measurement of cell apoptosis was carried out using Annexin V-FITC/propidium iodide (PI) double-staining kit (KeyGEN Biotech, Nanjing, China) as recommended by the manufacturers. A total event of 10,000 cells per sample was analyzed using a flow cytometer (BD Biosciences, Oxford, United Kingdom) with CellQuest software.

### Western Blot

Western blot analysis was implemented as previously described (Meng et al., 2015). Total protein was isolated from cultured HUVECs using the RIPA lysis buffer (Solarbio) and was quantified using BCA Protein Assay Kit (Thermo Fisher Scientific). Proteins (30 µg per lane) were resolved onto a 10% SDS–polyacrylamide gel and then transferred onto Clear Blot membrane-p (ATTO, Tokyo, Japan). The membranes were incubated with primary antibodies and horseradish peroxidase (HRP)–linked IgG secondary antibody (ab6721 or ab6728, Abcam, Cambridge, United Kingdom; dilution 1:10,000). The bound antibodies were detected by the enhanced chemiluminescence (ECL, Thermo Fisher Scientific) and analyzed using an image analyzer (LAS-4000, Fuji Photo Film, Minamiashigara, Japan). Primary antibodies against vascular endothelial growth factor (anti-VEGF, #MA1-16629; dilution 1:1,000), B-cell lymphoma 2 (anti-Bcl-2, #MA5-11757; dilution 1:50), Bcl-2–associated X protein (anti-Bax, #MA5-13994; dilution 1:100), and glyceraldehyde-3-phosphate dehydrogenase (anti-GAPDH, #AM4300; dilution 1:100) were purchased from Invitrogen (Toyko, Japan). Primary antibodies, including anti-Ki67 (#sc-23900; dilution 1:500), anti-poly (ADP-ribose) polymerases (anti-PARP, #sc-8007; dilution 1:1,000), and anti-Cleaved PARP (#sc-56196; dilution 1:1,000), were obtained from Santa Cruz Biotechnology (Santa Cruz, CA, United States). The FBXW7 antibody (anti-FBXW7, #ab192328; dilution 1:1,000) was obtained from Abcam.

### Bioinformatics and Dual-Luciferase Reporter Assay

The interacted miRNAs of circ_CLASP2 and miR-140-5p targets were predicted using the online starBase v.3 database (http://starbase.sysu.edu.cn/). The fragment of circ_CLASP2 harboring the miR-140-5p–pairing sites and the 3′-untranslated regions (3′-UTR) of FBXW7 were individually ligated into the pMIR-REPORT vector (Promega, Southampton, United Kingdom) to construct two reporter plasmids named circ_CLASP2-wt and FBXW7-wt. The mutations (circ_CLASP2-mut and FBXW7-mut) in the target region were generated using the QuikChange Mutagenesis Kit (Stratagene, Marcy L’Etoile, France) as per the manufacturing protocols. HUVECs (1 × 10^5^) were cotransfected with 100 ng of circ_CLASP2-wt, FBXW7-wt, circ_CLASP2-mut or FBXW7-mut, and 50 nM of miR-140-5p mimic or miR-NC mimic using Lipofectamine 3000. The *luciferase* activity was measured 24 h post-transfection by using the Promega dual-luciferase assays. Firefly *luciferase* activity was normalized to the activity of *Renilla luciferase*.

### Measurement of Nitric Oxide and Hydrogen Sulfide

The levels of NO and H_2_S were measured by using NO Colorimetric Assay Kit (Abcam) and H_2_S Assay Kit (Solarbio) as per the accompanying protocols. About 1 × 10^6^ treated HUVECs were used to measure NO and H_2_S production.

### Statistical Analysis

Data were presented as mean ± standard deviation (SD) from at least three biological repetitions. The GraphPad Prism 7 (GraphPad Software, La Jolla, CA, United States) was used to perform statistical analysis. A Student’s *t*-test was used to compare two groups, and the analysis among multiple groups was implemented using the one-way analysis of variance (ANOVA) with Tukey–Kramer post hoc test. Differences were considered significant at values of *p* < 0.05.

## Results

### Circ_CLASP2 Level was Downregulated in HG-Treated HUVECs

**FIGURE 1:**
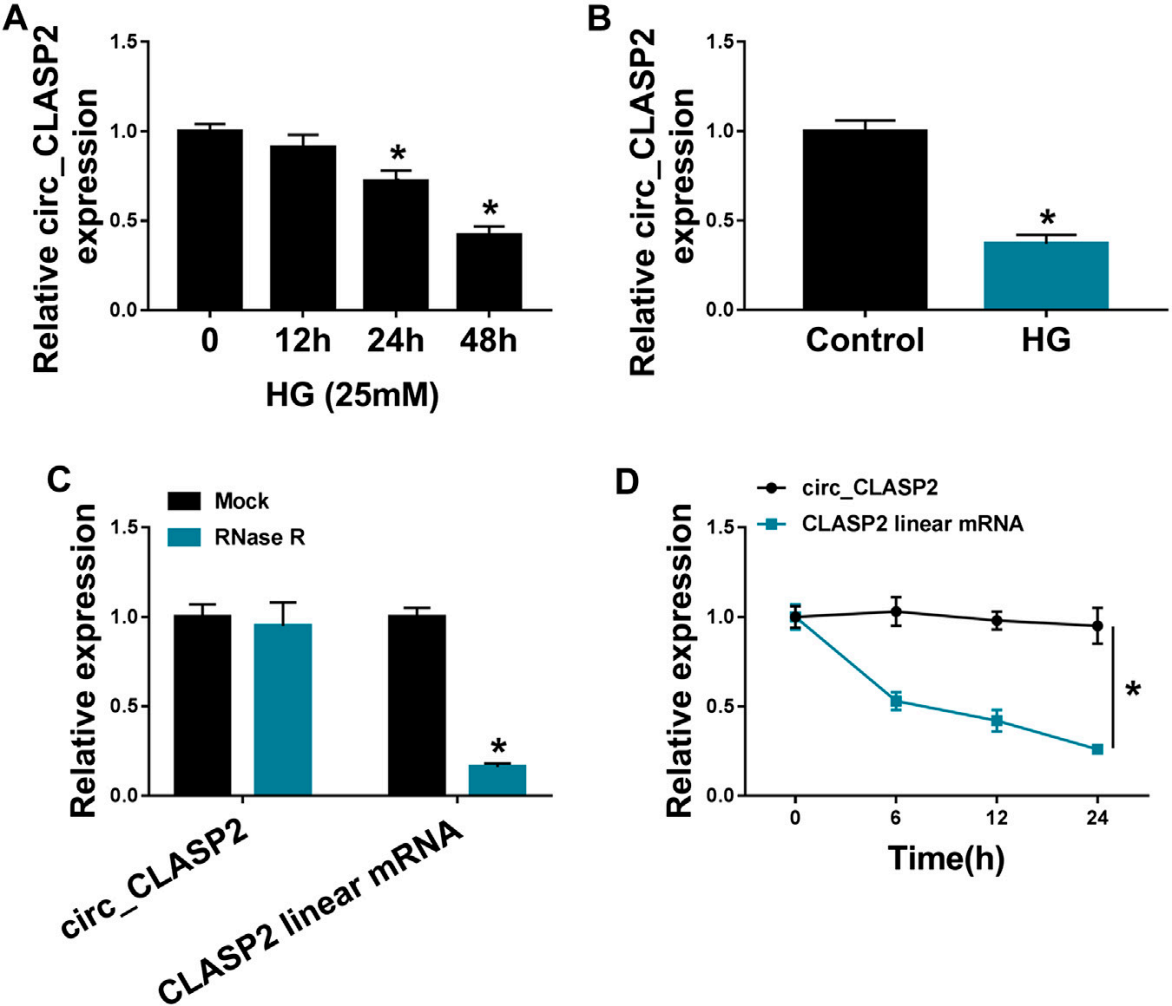
HG treatment reduced circ_CLASP2 expression in HUVECs. **(A)** HUVECs were treated with 25 mM of HG for 12, 24, and 48 h, and then circ_CLASP2 expression was assessed by qRT-PCR. **(B)** Circ_CLASP2 level was detected by qRT-PCR in HUVECs after the treatment of 25 mM of HG for 48 h. **(C)** Total RNA from HUVECs was incubated with RNase R, followed by the detection of circ_CLASP2 and CLASP2 linear mRNA by qRT-PCR. **(D)** The levels of circ_CLASP2 and CLASP2 linear mRNA were measured by qRT-PCR in HUVECs treated with actinomycin D. n = 3 independent biological replicates; data were presented as mean ± SD; *p < 0.05 by a two-tailed Student’s *t*-test or ANOVA followed by Tukey–Kramer post hoc test.

First, we validated the expression of circ_CLASP2 in HG-treated HUVECs. As demonstrated by qRT-PCR, in contrast to the negative control, circ_CLASP2 level was significantly reduced in HUVECs after HG exposure (Figures 1A,B). Then, we determined the stability of circ_CLASP2 using RNase R and actinomycin D assays. These results revealed that CLASP2 linear transcript was prominently decreased by RNase R treatment, and circ_CLASP2 was resistant to RNase R (Figure 1C). Moreover, the incubation of the cells with actinomycin D led to a striking reduction in the levels of CLASP2 linear mRNA, and circ_CLASP2 level was not affected in the assayed time frame (Figure 1D).

### Overexpression of circ_CLASP2 Promoted HUVEC Proliferation and Inhibited Apoptosis Under HG Conditions

**FIGURE 2:**
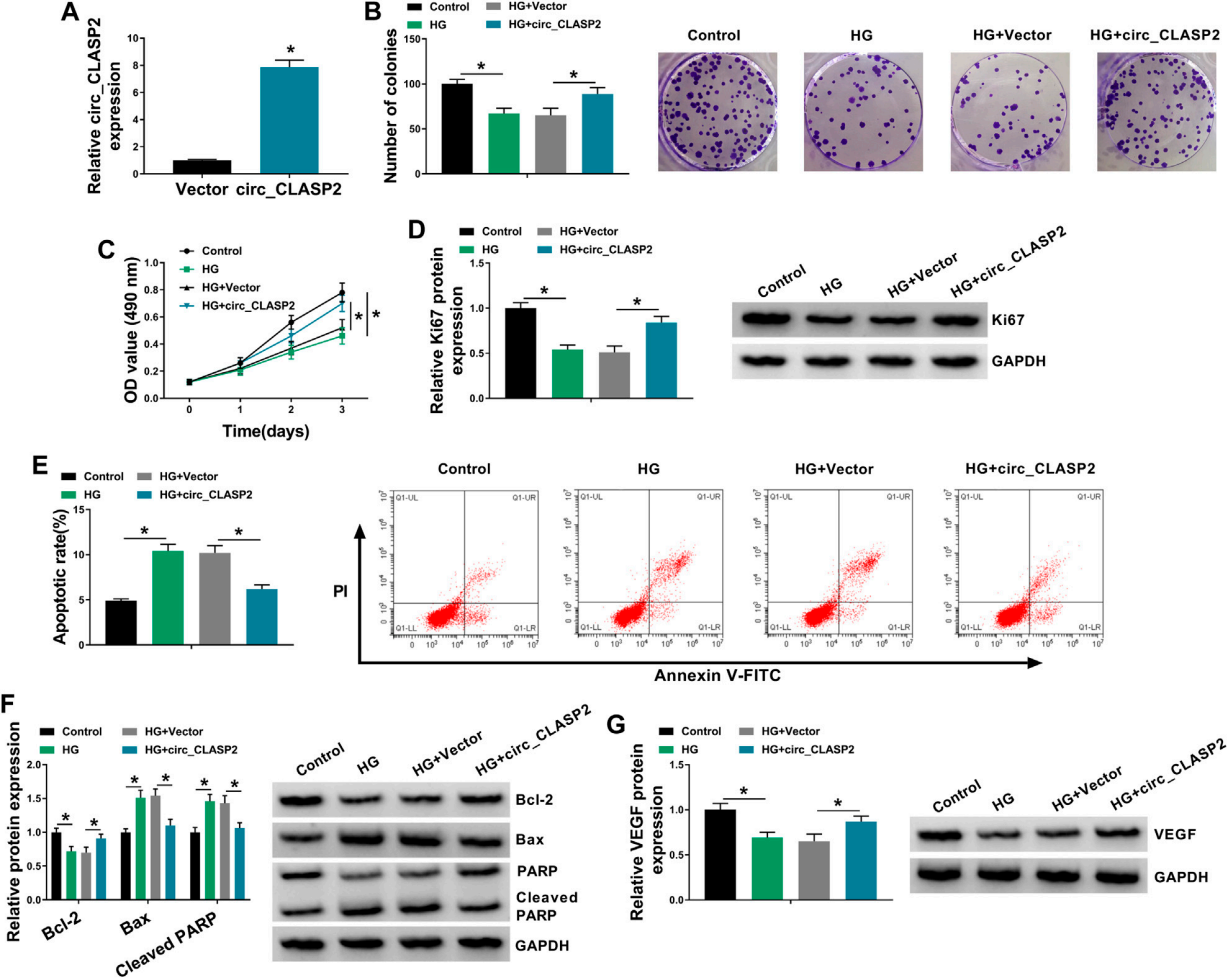
Circ_CLASP2 overexpression promoted proliferation and inhibited apoptosis in HUVECs under HG conditions. **(A)** Circ_CLASP2 expression was assessed by qRT-PCR in HUVECs transfected with Vector or circ_CLASP2. HUVECs were transfected with Vector or circ_CLASP2 and then treated with HG, followed by the measurement of cell colony formation using a colony formation assay **(B)**, cell proliferation by MTT assay **(C)**, Ki67 level by Western blot **(D)**, cell apoptosis by flow cytometry **(E)**, Bcl-2, Bax and Cleaved PARP levels **(F),** and VEGF expression **(G)** by Western blot. Vector: negative control vector, circ_CLASP2: circ_CLASP2 overexpression vector. n = 3 independent biological replicates; data were presented as mean ± SD; *p < 0.05 by a two-tailed Student’s *t*-test or ANOVA followed by Tukey–Kramer post hoc test.

To observe the influence of circ_CLASP2 on HG-treated HUVECs, we manipulated its expression using the overexpression vector. qRT-PCR data revealed that in comparison to the negative group, circ_CLASP2 expression was significantly elevated by the transfection of circ_CLASP2 overexpression vector (Figure 2A). The results of colony formation and MTT assays showed that cell colony formation and proliferation were markedly repressed by HG treatment (Figures 2B,C). Western blot analyses revealed that HG treatment resulted in decreased expression of proliferating marker Ki67 in HUVECs (Figure 2D), supporting the inhibition of HG on cell proliferation. Flow cytometry data demonstrated that cell apoptosis was highly enhanced by HG treatment (Figure 2E). Moreover, HG treatment led to a distinct reduction in anti-apoptotic protein Bcl-2 expression and a clear elevation in apoptosis markers Bax and Cleaved PARP levels (Figure 2F). Additionally, the treatment of HG triggered a diminution in VEGF expression (Figure 2G).

### Circ_CLASP2 Directly Interacted with miR-140-5p Through Binding to miR-140-5p

**FIGURE 3:**
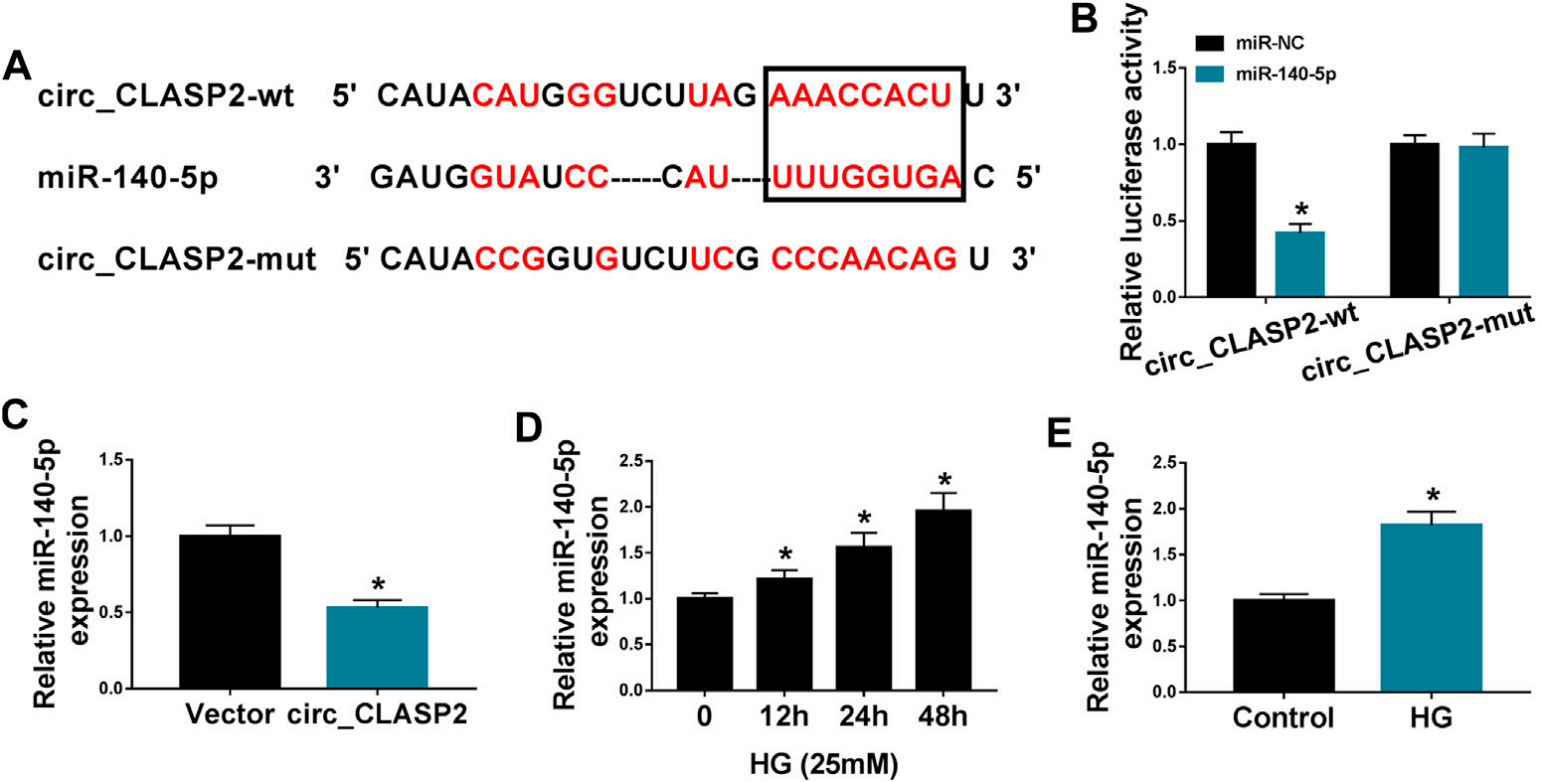
Circ_CLASP2 directly interacted with miR-140-5p in HUVECs. **(A)** Schematic of the complementary sequence for miR-140-5p within circ_CLASP2 identified by the starBase v.3 database and the mutant of the seed sequence. **(B)** Relative luciferase activity was detected in HUVECs cotransfected with circ_CLASP2 wild-type reporter (circ_CLASP2-wt) or circ_CLASP2 mutant-type reporter (circ_CLASP2-mut) and miR-140-5p mimic or miR-NC mimic. **(C)** MiR-140-5p expression was evaluated by qRT-PCR in HUVECs transfected with Vector or circ_CLASP2. Vector: negative control vector, circ_CLASP2: circ_CLASP2 overexpression vector. **(D,E)** MiR-140-5p expression was tested by qRT-PCR in HUVECs after HG treatment. n = 3 independent biological replicates; data were presented as mean ± SD; *p < 0.05 by a two-tailed Student’s *t*-test or ANOVA followed by Tukey–Kramer post hoc test.

To determine the mechanism by which circ_CLASP2 regulated HUVEC proliferation and apoptosis under HG conditions, we used the online starBase v.3 database to help identify the interacted miRNAs of circ_CLASP2. A potential complementary sequence for miR-140-5p was discovered within circ_CLASP2 (Figure 3A). To verify this, we cloned the sequence of circ_CLASP2 into a luciferase plasmid and mutated the complementary sequence. With circ_CLASP2 reporter construct, and miR-140-5p overexpression caused a significant downregulation in luciferase activity (Figure 3B). When the target sequence was mutated, the downregulation of miR-140-5p in luciferase was strongly abolished under the same conditions (Figure 3B), indicating the validity of the complementary sites for interaction. Moreover, in contrast to the negative control, miR-140-5p expression was remarkably decreased by the upregulation of circ_CLASP2 (Figure 3C). Additionally, the data of qRT-PCR demonstrated that miR-140-5p level was prominently elevated in HUVECs after HG treatment (Figures 3D,E).

### MiR-140-5p Mediated the Regulation of circ_CLASP2 Overexpression on HUVEC Proliferation and Apoptosis Under HG Conditions

**FIGURE 4:**
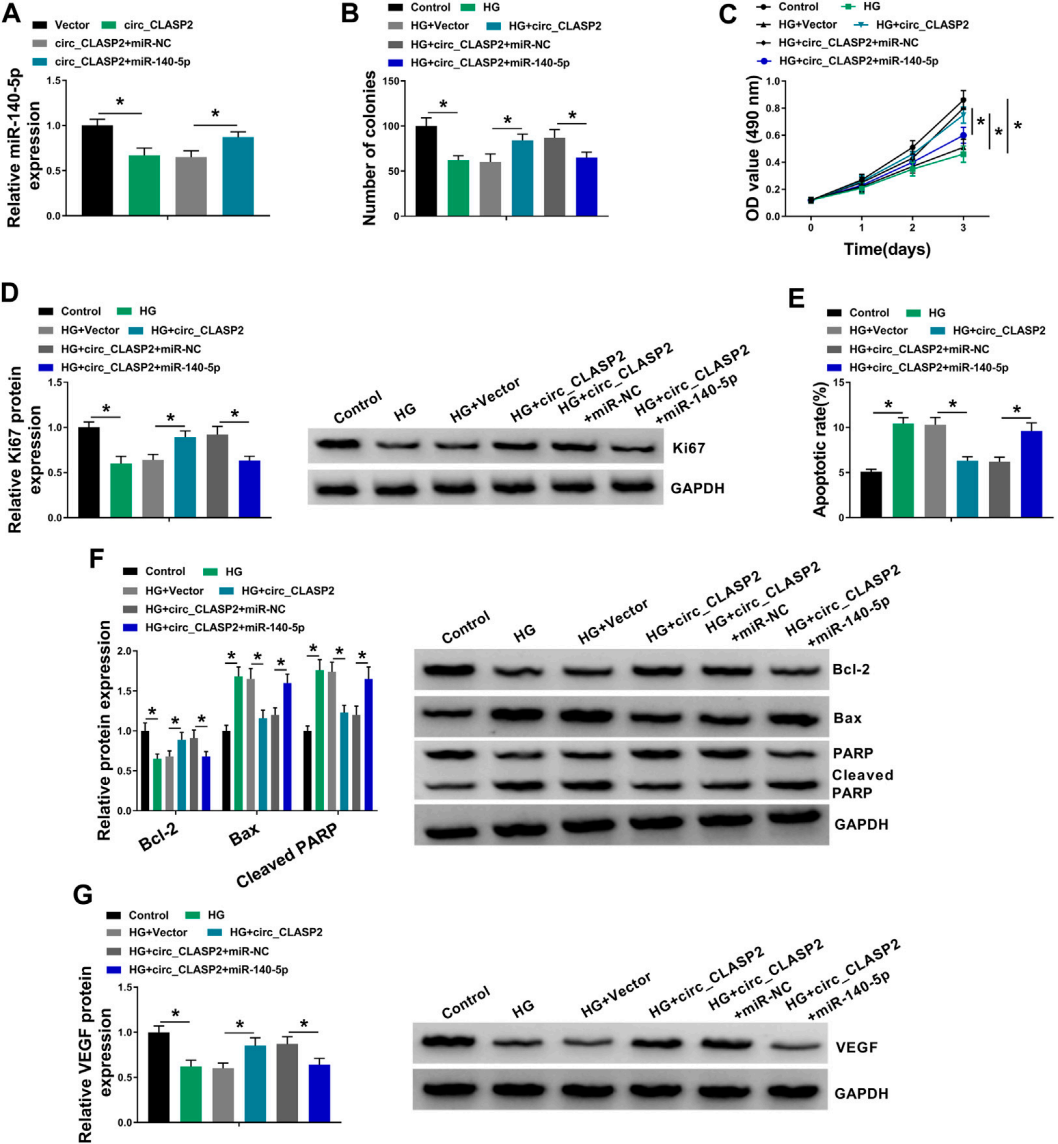
Overexpression of circ_CLASP2 regulated proliferation and apoptosis of HG-treated HUVECs by miR-140-5p. **(A)** MiR-140-5p expression was tested by qRT-PCR in HUVECs transfected with Vector, circ_CLASP2, circ_CLASP2+miR-NC mimic, or circ_CLASP2+miR-140-5p mimic. HUVECs were transfected with Vector, circ_CLASP2, circ_CLASP2+miR-NC mimic, or circ_CLASP2+miR-140-5p mimic before HG treatment, followed by the determination of cell colony formation using a colony formation assay **(B)**, cell proliferation by MTT assay **(C)**, Ki67 level by Western blot **(D)**, cell apoptosis by flow cytometry **(E)**, Bcl-2, Bax and Cleaved PARP levels **(F)**, and VEGF expression **(G)** by Western blot. Vector: negative control vector, circ_CLASP2: circ_CLASP2 overexpression vector. n = 3 independent biological replicates; data were presented as mean ± SD; *p < 0.05 by ANOVA followed by Tukey–Kramer post hoc test.

Then, we investigated whether circ_CLASP2 regulated cell proliferation and apoptosis of HG-treated HUVECs by miR-140-5p. As shown in Figure 4A, circ_CLASP2 overexpression-mediated miR-140-5p diminishment was dramatically reversed by the cotransfection of miR-140-5p mimic in HUVECs. Moreover, in comparison to the negative control, circ_CLASP2 overexpression-mediated pro-colony formation (Figure 4B), pro-proliferation (Figures 4C,D), and anti-apoptosis (Figures 4E,F) were significantly abrogated by the restored expression of miR-140-5p in HUVECs under HG conditions. Furthermore, the results of Western blot revealed that miR-140-5p level restoration resulted in decreased VEGF expression, which was upregulated by circ_CLASP2 overexpression in HG-treated HUVECs (Figure 4G).

### Circ_CLASP2 Protected Against FBXW7 Repression Through Sponging miR-140-5p

**FIGURE 5:**
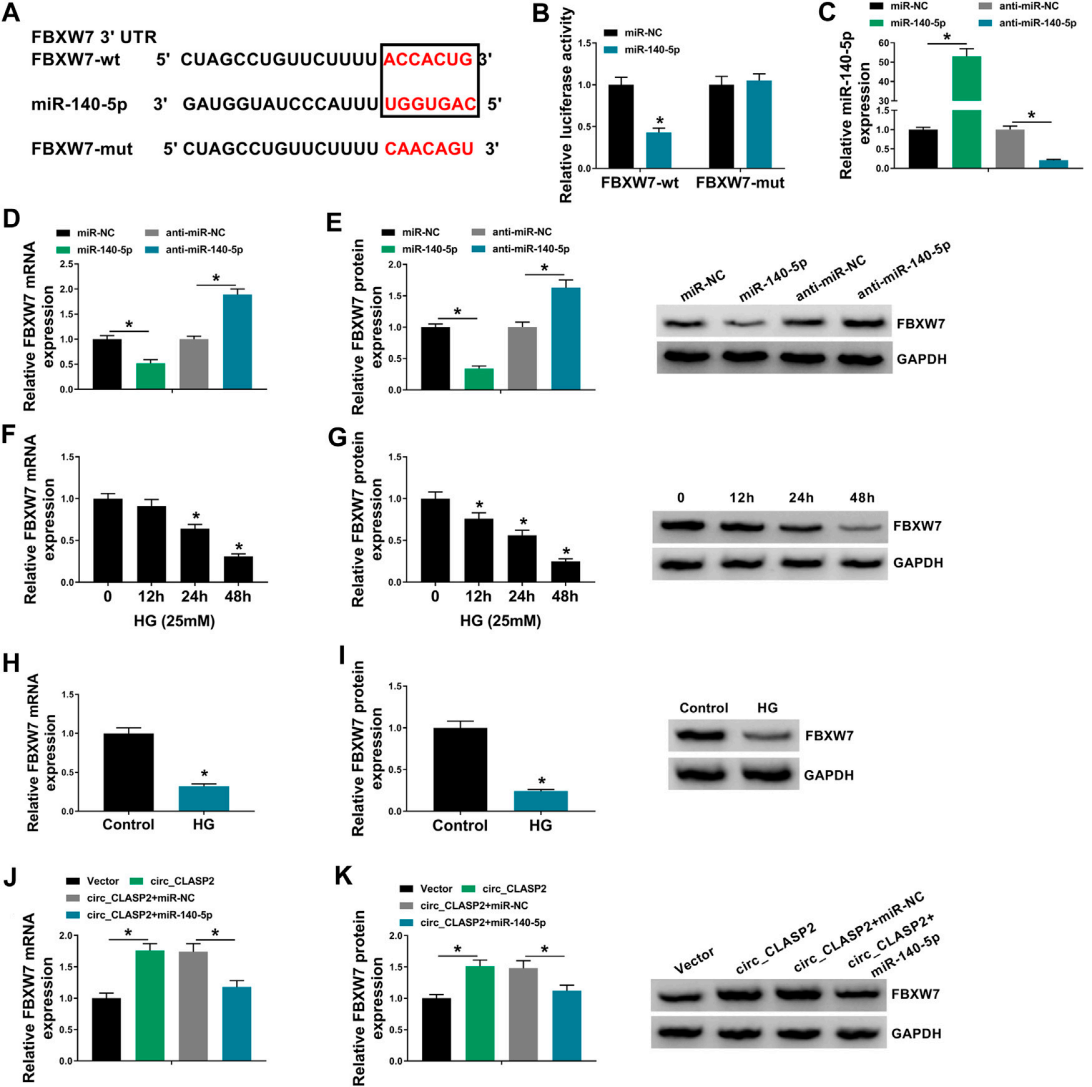
Circ_CLASP2 mediated FBXW7 expression through sponging miR-140-5p. **(A)** Schematic of illuminating the target sequence for miR-140-5p within FBXW7 3′-UTR and the mutant in the target region. **(B)** Relative luciferase activity was detected in HUVECs transfected with FBXW7 3′-UTR luciferase reporter (FBXW7-wt) or the mutant in the miR-140-5p–binding sites (FBXW7-mut) and miR-140-5p mimic or miR-NC mimic. MiR-140-5p expression **(C)**, FBXW7 mRNA level **(D)**, and FBXW7 protein expression **(E)** were assessed by qRT-PCR or Western blot assay in HUVECs transfected with miR-NC mimic, miR-140-5p mimic, anti-miR-NC, or anti-miR-140-5p. **(F–I)** FBXW7 mRNA and protein levels were detected in HUVECs after HG treatment. **(J,K)** FBXW7 mRNA and protein levels were tested in HUVECs transfected with Vector, circ_CLASP2, circ_CLASP2+miR-NC mimic, or circ_CLASP2+miR-140-5p mimic. Vector: negative control vector, circ_CLASP2: circ_CLASP2 overexpression vector. n = 3 independent biological replicates; data were presented as mean ± SD; *p < 0.05 by a two-tailed Student’s *t*-test or ANOVA followed by Tukey–Kramer post hoc test.

Using starBase v.3 software, a putative target sequence for miR-140-5p was predicted within the 3′-UTR of FBXW7 (Figure 5A). Transient transfection of miR-140-5p mimic, but not the scrambled negative sequence, markedly reduced the luciferase activity of FBXW7 3′-UTR luciferase reporter (Figure 5B). However, little reduction was observed in luciferase of the site-directed mutant in the presence of miR-140-5p mimic (Figure 5B). qRT-PCR assays revealed that in contrast to their counterparts, miR-140-5p expression was significantly elevated by miR-140-5p mimic and decreased by anti-miR-140-5p in HUVECs (Figure 5C). Moreover, the data of qRT-PCR and Western blot analyses showed that FBXW7 mRNA and protein levels were prominently reduced by miR-140-5p overexpression, while they were highly increased when miR-140-5p silencing (Figures 5D,E), indicating that the miR-140-5p–binding sites were functional. Additionally, FBXW7 expression was significantly downregulated in HG-treated HUVECs (Figures 5F–I). Furthermore, FBXW7 mRNA and protein levels were strikingly elevated when circ_CLASP2 was overexpressed, whereas this effect was remarkably abolished by the transfection of miR-140-5p mimic in HUVECs (Figures 5J,K).

### MiR-140-5p Regulated HUVEC Proliferation and Apoptosis Under HG Conditions by FBXW7

**FIGURE 6:**
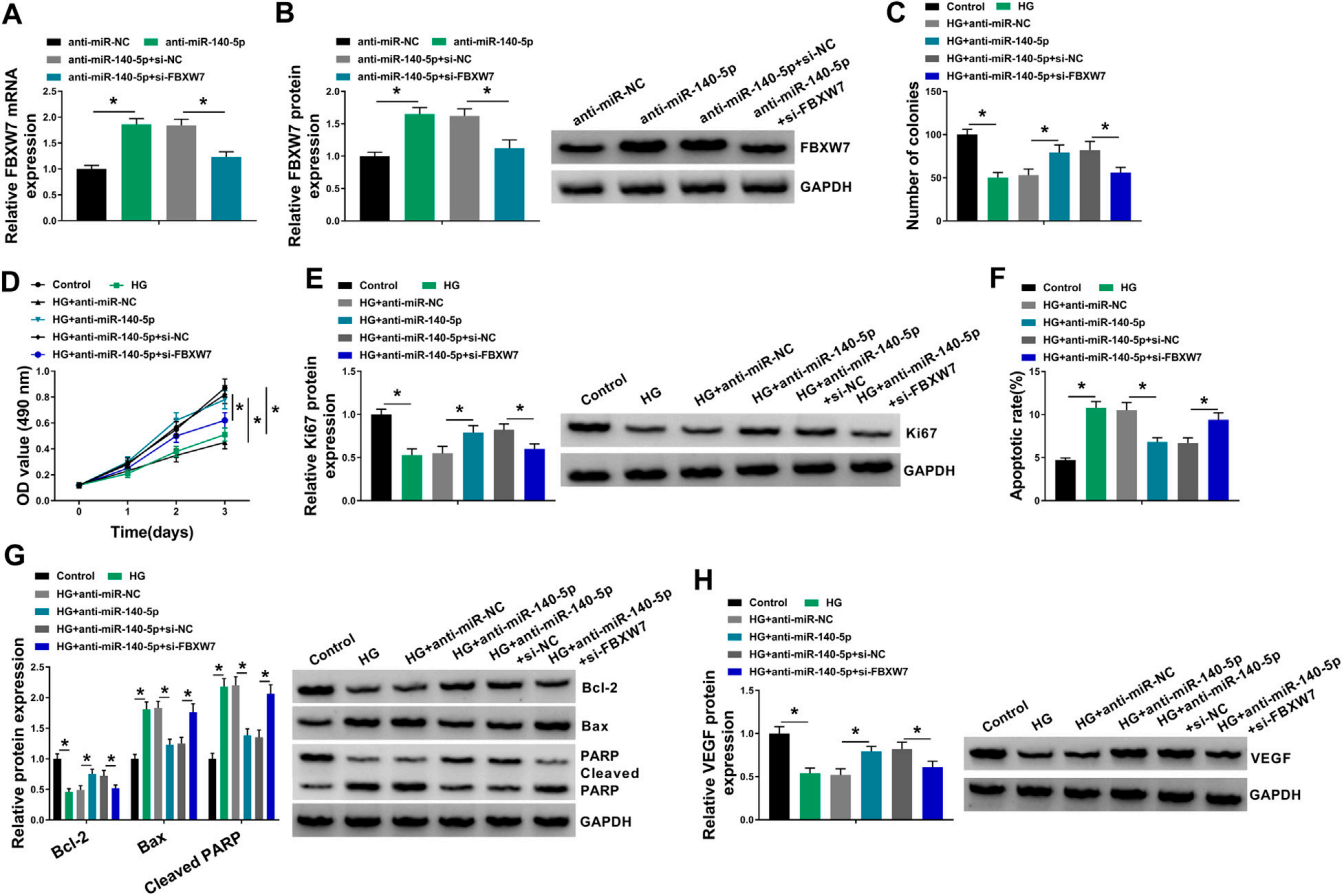
MiR-140-5p regulated HUVEC proliferation and apoptosis under HG conditions by FBXW7. **(A,B)** FBXW7 mRNA and protein levels were measured by qRT-PCR or Western blot assay in HUVECs transfected with anti-miR-NC, anti-miR-140-5p, anti-miR-140-5p + si-NC, or anti-miR-140-5p + si-FBXW7. HUVECs were transfected with anti-miR-NC, anti-miR-140-5p, anti-miR-140-5p + si-NC, or anti-miR-140-5p + si-FBXW7 before HG treatment, followed by the determination of cell colony formation using a colony formation assay **(C)**, cell proliferation by MTT assay **(D)**, Ki67 level by Western blot **(E)**, cell apoptosis by flow cytometry **(F)**, Bcl-2, Bax and Cleaved PARP levels **(G)**, and VEGF expression **(H)** by Western blot. *p < 0.05. n = 3 independent biological replicates; data were presented as mean ± SD; *p < 0.05 by ANOVA followed by Tukey–Kramer post hoc test.

To provide further mechanistic insight into the correlation between miR-140-5p and FBXW7 on HG-treated HUVECs, the cells were cotransfected with anti-miR-140-5p and si-FBXW7. In comparison to the negative control, the promotional impact of miR-140-5p silencing on FBXW7 expression was prominently reversed by si-FBXW7 transfection (Figures 6A,B). Functional experiments’ results demonstrated that miR-140-5p silencing led to an obvious enhancement in cell colony formation (Figure 6C), cell proliferation (Figures 6D,E), and a striking repression in cell apoptosis (Figures 6F,G), as well as a significant augment in VEGF expression (Figure 6H) in HUVECs under HG conditions. Nevertheless, these effects of miR-140-5p silencing were remarkably abrogated by the stored expression of FBXW7 in HG-treated HUVECs (Figures 6C–H).

## Discussion

Diabetes mellitus is a leading health problem all over the world (American Diabetes Association, 2013). The management of diabetes has mainly focused on the control of hyperglycemia, which is a contributing factor for its macrovascular complications (Marcovecchio et al., 2011). Increasing studies have shown that a crowd of circRNAs can potentially function as biomarkers for the detection, treatment, and prevention of endothelial dysfunction (Liu et al., 2017; Shan et al., 2017; Liu et al., 2019). In the current study, we focused on the function of circ_CLASP2 on HG-induced HUVEC dysfunction.

It was reported that circ_CLASP2 expression was prominently decreased in HUVECs under HG exposure (Jin, Wang, 2019). To validate this, we used HG to treat HUVECs for various time points, and our data supported the downregulation of circ-CLASP2 in HG-treated HUVECs. Consistent with our findings, HG exposure has been indicated to weaken the expression of circ_001175 and circHIPK3 (Cao et al., 2018; Pei et al., 2018). Due to the covalently closed loop structures, circRNAs are unusually stable and resistant to RNase R (Du et al., 2017). As previously reported for other circRNAs (Li et al., 2015; Wang et al., 2018), circ_CLASP2 was stable and its level did not reduce in the assayed time frame using RNase R and actinomycin D assays. Subsequently, for the first time, our data uncovered that the elevated expression of circ_CLASP2 enhanced HUVEC proliferation and suppressed apoptosis under HG exposure. VEGF, an angiogenic factor of ECs, has been underscored to implicate in ECs proliferation and apoptosis induced by HG, and VEGF restoration might be beneficial for diabetic endothelial dysfunction (Kim et al., 2002; Yang et al., 2008). We found that circ_CLASP2 overexpression led to an increase in VEGF expression in HG-treated HUVECs. In a word, the overexpression of circ_CLASP2 performed a protective role in HG-induced HUVEC dysfunction.

CircRNAs are widely recognized as miRNA sponges to protect against gene expression repression (Kulcheski et al., 2016). Using the online starBase v.3 database, miR-140-5p was predicted as a potentially interacted miRNA of circ_CLASP2, which was validated by further experiments. Accumulating evidence has demonstrated that miR-140-5p regulates the development of a series of human cancers, such as bladder cancer, gastric cancer, and triple-negative breast cancer (Wang et al., 2016; Fang et al., 2017; Yu et al., 2019). MiR-140-5p was also reported to weaken the development of osteoarthritis (Tao et al., 2017; Wang et al., 2020). Moreover, Sun et al. highlighted that miR-140-5p hindered angiogenesis in an *in vitro* ischemia model through the regulation of VEFGA expression (Sun et al., 2016). These researches described as above provided the possibility for the important involvement of miR-140-5p in diabetic endothelial dysfunction. In the present work, we discovered that HG exposure elevated miR-140-5p expression in HUVECs, in agreement with former work (Silambarasan et al., 2016). Furthermore, we were first to uncover that miR-140-5p deficiency promoted cell proliferation and hampered apoptosis in HG-treated HUVECs. Previous literature had reported that several other miRNAs, such as miR-137 and miR-221, were significantly upregulated by HG exposure in HUVECs (Li et al., 2009; Li et al., 2016). Li et al. underscored that the upregulation of miR-221 mitigated HUVEC migration (Li et al., 2009). Li and colleagues underscored that miR-137 knockdown relieved HG-induced cell viability reduction and apoptosis enhancement via targeting AMP-activated protein kinase α1 (AMPKα1) (Li et al., 2016). More interestingly, for the first time, we highlighted that the regulation of circ_CLASP2 overexpression on HG-induced HUVEC dysfunction was mediated by miR-140-5p.

Then, we used the computational method to help identify the targets of miR-140-5p. Among these candidates, we selected FBXW7 for further exploration because of its beneficial role in glucose homeostasis (Zhao et al., 2018). Shao et al. highlighted that FBXW7 was implicated in the regulation of transthyretin on neovascularization in diabetic retinopathy (Shao et al., 2019). Moreover, FBXW7 was reported as a crucial regulator of endothelial barrier function and endothelium vasculature (Izumi et al., 2012; Pronk et al., 2019). We verified that miR-140-5p directly targeted FBXW7 in HUVECs, and miR-140-5p regulated HG-induced cell dysfunction by FBXW7. Furthermore, we were first to uncover that circ_CLASP2 mediated FBXW7 expression through sponging miR-140-5p. Abnormal expression of NO and H_2_S has been widely reported in HG-treated human endothelial cells, and they are involved in endothelial dysfunction induced by HG (Ho et al., 1999; Xue et al., 2020). Our data showed that the increased expression of circ_CLASP2 could reverse the impact of NO and H_2_S production of HG in HUVECs (Supplementary Figure S1). These findings suggested that circ_CLASP2 might regulate HG-induced endothelial dysfunction by mediating NO and H_2_S production. A future challenge will be to identify whether circ_CLASP2 regulates NO and H_2_S production in HG-induced HUVECs by the miR-140-5p/FBXW7 axis. In the current work, we used HG-treated HUVECs as the model of the *in vivo* ECs under hyperglycemia. To identify groups of circRNAs for macrovascular injury in specific organs, further investigations should be carried out using the respective cells derived from the blood vessels to the target organs.

In conclusion, our present research suggested that the overexpression of circ_CLASP2 protected HUVEC from HG-induced dysfunction by regulating FBXW7 expression by sponging miR-140-5p. Our findings illumined a novel mechanism of the circ_CLASP2/miR-140-5p/FBXW7 axis in diabetes-associated endothelial dysfunction.

## Data Availability

The original contributions presented in the study are included in the article/Supplementary Material, further inquiries can be directed to the corresponding author.
