# Supplementary material for: Circ_CLASP2 Regulates High Glucose-Induced Dysfunction of Human Endothelial Cells Through Targeting miR-140-5p/FBXW7 Axis
Source: Front Pharmacol. 2021 Mar 11;12:594793. doi: 10.3389/fphar.2021.594793 (PMC7990784; doi:10.3389/fphar.2021.594793)
Supplement: Supplementary file 1 [file datasheet1.docx]

**Supplement Figure S1. The impact of circ_CLASP2 on NO and H_2_S expression levels in HG-treated HUVECs.** HUVECs were transfected with Vector or circ_CLASP2 and then exposed to HG for 48 h, followed by the measurement of NO (A) and H_2_S (B) concentrations using the assay kits. n = 3 independent biological replicates; data were presented as mean ± SD; **P* < 0.05 by ANOVA followed by Tukey-Kramer post hoc test.
