# Supplementary figures and images for: Circ_CLASP2 Regulates High Glucose-Induced Dysfunction of Human Endothelial Cells Through Targeting miR-140-5p/FBXW7 Axis
Source: Front Pharmacol. 2021 Mar 11;12:594793. doi: 10.3389/fphar.2021.594793 (PMC7990784; doi:10.3389/fphar.2021.594793)

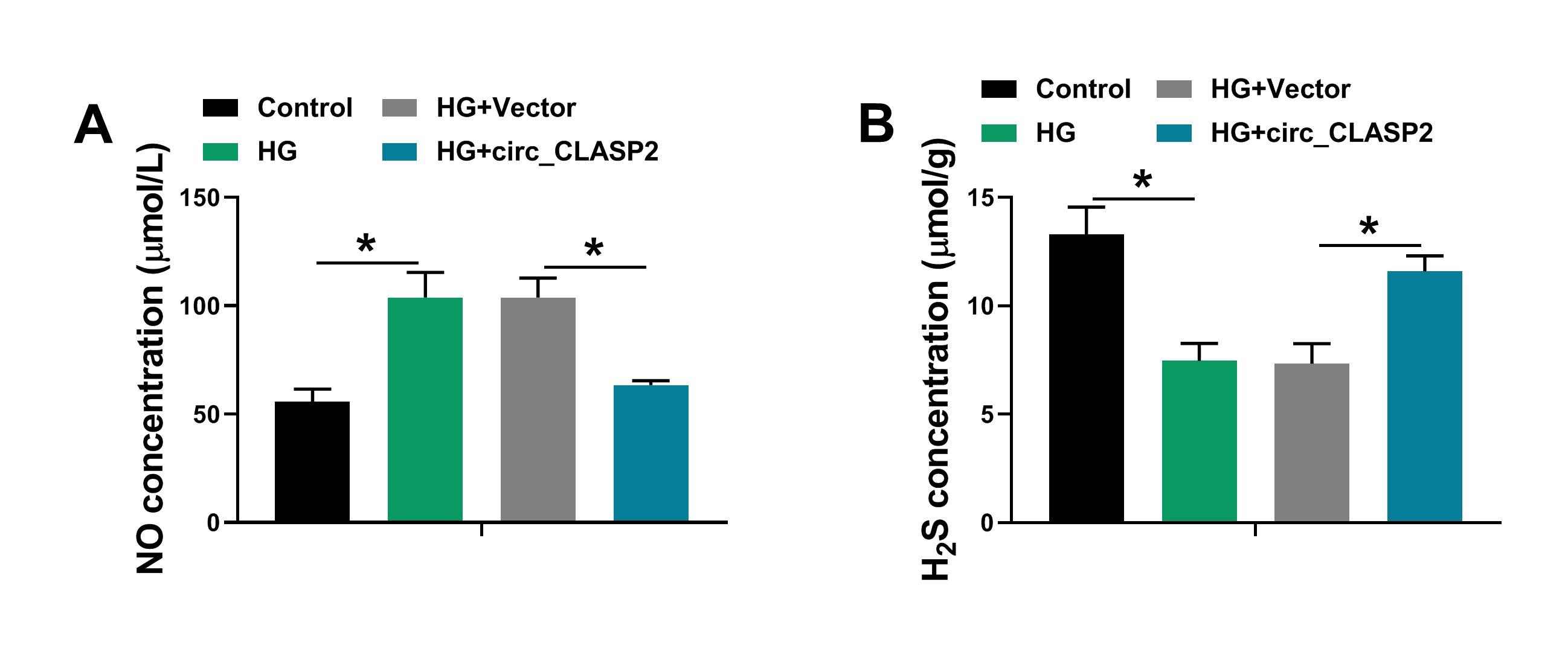

Supplement: Supplementary file 2 [file image1.tif]
